# Temporal Effects of Lipid Oversupply on Energy Metabolism and Mitochondrial Homeostasis in Hepatocytes

**DOI:** 10.7150/ijms.104128

**Published:** 2025-07-28

**Authors:** Cheng-Chieh Chuang, Ying-Hao Chen, Fang-Yeh Chu, Ching-Ping Yang, Hsiang-Ling Ho, Fu-Pang Chang, Yu-Li Lo, Chun-Jung Chen, Yih-Hsin Chang

**Affiliations:** 1 Department of Biotechnology and Laboratory Science in Medicine, National Yang Ming Chiao Tung University, Taiwan.; 2 Department of Clinical Pathology, Far Eastern Memorial Hospital, New Taipei City 220; Taiwan.; 3 Graduate School of Biotechnology and Bioengineering, Yuan Ze University, Taoyuan 320; Taiwan.; 4 Department of Medical Laboratory Science and Biotechnology, Yuanpei University 300; Taiwan.; 5 School of Medical Laboratory Science and Biotechnology, Taipei Medical University, Taipei 110; Taiwan.; 6 Department of Pathology and Laboratory Medicine, Taipei Veterans General Hospital, Taipei 112; Taiwan.; 7 Department and Institute of Pharmacology, National Yang Ming Chiao Tung University, Taipei 112; Taiwan.; 8 Department of Medical Research, Taichung Veterans General Hospital, Taichung 407; Taiwan.; 9 Department of Medical Laboratory Science and Biotechnology, China Medical University, Taichung 404; Taiwan.

**Keywords:** obesity, lipid oversupply, energy metabolism, mitochondrial homeostasis, hepatocytes

## Abstract

Obesity is closely associated with multiple metabolic disorders such as non-alcoholic fatty liver disease (NAFLD). Patients with NAFLD are susceptible to develop irreversible life-threatening diseases, however, the evolution concerning mitochondrial and metabolic alterations during NAFLD development and progression remain elusive. This study focused on uncovering the sequential events of energy metabolism and mitochondrial homeostasis of hepatocytes under the environment of lipid oversupply by *in vitro* and *in vivo* strategies. Long-chain fatty acid (FA) synthesis and lipid storage were first induced by providing hepatocytes with sufficient energy source, followed by suppressed glucose metabolic efficiency and decreased mitochondrial mass. Intriguingly, distinctive features of hepatic cancer cells in response to FA oversupply were characterized. Insulin signaling and glucose uptake were rapidly deterred while lipid β-oxidation was significantly boosted. Enhanced mitochondrial biogenesis was identified as compensatory feedback for mitochondrial dysfunction. FA-induced mitophagy, cell morphological transition and higher N-cadherin expression potentiates epithelial-mesenchymal transition (EMT) which confers the cells with higher plasticity and accelerates NAFLD progression to irreversible hepatic diseases. This study provides evidence elucidating the temporal events caused by FA oversupply, moreover, delineates the facilitative role of excess nutrients in shaping the environment for lipid-laden hepatocytes to acquire malignant traits. Given the rapidly increasing global prevalence of metabolic disorders and the heterogeneous manifestations exhibited by NAFLD during disease progression, better understanding of the sequential events caused by FA overload aids in identifying promising targets and developing tailor-made treatment protocol according to individual disease status and conditions.

## Introduction

Obesity is closely associated with multiple metabolic disorders 1, 2. Insulin resistance, defined by impaired insulin signaling which results in diminished glucose uptake and dysregulated energy metabolism, is frequently preceded the onset of glucose intolerance and type 2 diabetes mellitus (T2DM). The major characteristics of T2DM is actually a manifestation of underlying intertwined disorders, leading to hepatic steatosis or non-alcoholic fatty liver disease (NAFLD), one of the most prevalent liver diseases due to the rapid increasing obesity population.

Hepatocytes are metabolically active cells containing ample mitochondria which generate energy by oxidative phosphorylation (OXPHO) and lipid oxidation 3. Mitochondria consistently adjust their status via biogenesis, fusion and fission (collectively designated as mitochondrial dynamics) to meet cellular energy needs and maintain mitochondrial homeostasis (mitostasis) 4. Among the mitostasis-controlling machinery, mitofusin 1 (MFN1), MFN2 and optic atrophy 1 (OPA1) are critical proteins mediating fusion 5-7 while dynamin-related protein-1 (DRP1) and mitochondrial fission protein-1 (FIS1) play important roles in fission 5, 8. Mitophagy also takes part in the maintenance of mitostasis, which is mainly modulated by mitochondrial quality control keeper PTEN-induced kinase 1 (PINK1) 9. Mitochondrial dysfunction is known to aggravate NAFLD progression 10.

Liver transforms excess intracellular free fatty acids (FFAs) or glucose into triglycerides (TGs) and thus stores TGs in lipid droplets (LDs). Loading and storing excessive amount of TGs as LDs in liver triggers metabolic reprogramming which leads to dysfunctional metabolism, oxidative stress and mitochondria 11-13. As high as 76% of people with obesity are suffering NAFLD 14, and about half NAFLD individuals have obesity and T2DM comorbidity. Without appropriate intervention, long-term NAFLD patients are susceptible to develop irreversible life-threatening disorders including nonalcoholic steatohepatitis (NASH), fibrosis, cirrhosis, and hepatocellular carcinoma (HCC) 15,16. In this context, developing effective therapeutic strategy for ameliorating hepatic lipid overload is the best policy to prevent NAFLD progression despite appropriate lifestyle interventions can effectively alleviate or reverse symptoms 17. In particular, non-compliance of NAFLD patients highlights the critical need of appropriate strategies to prevent disease progression and subsequent consequences 17.

Our previous study 18 reveals that presenilin-associated rhomboid-like protein (PARL)-mediated cleavage of full-length PINK1 (f-PINK1) into small PINK1 fragment (s-PINK1) plays critical roles in adipocyte differentiation (adipogenesis), and therefore, determines body lipid reservoir. This finding provides a mutual link concerning lipid deposits in adipose tissue and liver. Accordingly, mitochondrial quality control harbors the potential to be a therapeutic target for obesity and NAFLD 19.

Nevertheless, further investigations are needed to disclose the temporal events of mitochondrial alterations and metabolic changes induced by lipid oversupply 20. In this context, the present study focused on temporally examining mitostasis-controlling machinery and lipid metabolism of hepatocytes under FA oversupply by *in vitro* and *in vivo* strategies to elucidate the sequential events of mitochondrial homeostasis and energy metabolism in response to FA overload.

## Materials and Methods

### Reagents

Reagents were obtained from the following sources: Antibodies against acetyl coenzyme A carboxylase (ACC), phospho-Ser^79^ ACC (pACC), CCAAT-enhancer-binding protein-alpha (C/EBPα), peroxisome proliferator-activated receptor-gamma (PPARγ), fatty acid-binding protein 4 (FABP4), Akt, phospho-Ser^473^ Akt (pAkt), Parkin, and β-actin form Cell Signaling Technology (Danvers, MA, USA); antibodies against fatty acid synthase (FAS) from BD Biosciences; anti-Drp1 from Novus Biologicals (Littleton CO, USA); antibodies against PARL, LC3B, carnitine palmitoyl transferase 1A (CPT1A), diacylglyceride acyltransferase 2 (DGAT2), MFN2, and PARL from GenTex, Inc. (Irvine, CA, USA); antibodies against PINK1, voltage-dependent anion-selective channel (VDAC) and Parkin from Abcam (San Francisco, CA, USA); ECL reagent from Calbiochem (Merck Millipore, Billerica, MA, USA); insulin, palmitic acid (PA) and oleic acid (OA) from Sigma (St. Louis, MO, USA); Trizol Reagent and Applied Biosystems SYBR Green Realtime PCR Master Mix from Life Technology (Carlsbad, CA, USA).

### Cell culture and treatments

BNL (from Bioresource Collection and Research Center, Taiwan; BCRC Number 60216) and HepG2 (BCRC number RM60025) hepatocytes were cultured in DMEM (HyClone) containing 1% penicillin, streptomycin and 10% fetal bovine serum at 37^o^C. For insulin treatment, after 4 hours of serum starvation, cells were treated with 100 nM insulin for the time indicated. For OA and/or PA treatment, cells were cultured with differential doses of OA and/or PA (0.1, 0.25 and 0.35 mM) as the time indicated. Oil-Red O (ORO) staining was performed to measure intracellular lipid contents.

### RNA extraction and RT-PCR

After being reverse-transcribed, PPARγ coactivator 1 alpha (PGC1α) was amplified by 30 cycles of PCR reaction using specific primers (5'-AGTTTTTGGTGAAATTGAGGAAT-3' and 5'-TCATACTTGCTCTTGGTGGAAGC-3') and analyzed by StepOnePlus™ real-time PCR system (Applied Biosystems, US).

### Western blot

Proteins in total cell lysates were resolved by SDS-PAGE, electrostransferred to PVDF membrane, incubated with specific primary antibodies and HRP-conjugated secondary antibodies (ZYMED Laboratories Inc & NEN, Boston, USA), visualized by ECL and quantitated by densitometry.

### Glucose uptake fluorometric assay

Cells were first incubated with glucose-free KRPH buffer for 3 h, treated with IL-4 and/or insulin for 20 min, fed with 100 µmol/L 2-[N-(7-nitrobenz-2-oxa-1,3-diazol-4-yl)amino]-2-deoxy-D-glucose (2-NBDG) for 10 min, and terminated by ice-cold KRPH buffer containing 10 mM glucose. Cells were washed, lysed, and intracellular fluorescence intensity was measured (485⁄540 nm, Infinite 200).

### Cell fractionation and mitochondrial isolation

Cell fractionation and mitochondrial isolation were conducted as described 18. In brief, cells were homogenized with mitochondria isolation buffer after the indicated treatment, subjected to sequential differential centrifugation (600 g for 10 min, then 7,000 g for 10 min) at 4^o^C to collect cytosol supernatant and intact mitochondria in the sediment. Mitochondrial pellet was resuspended in lysis buffer and subjected to centrifugation (12,000 g for 15 min) to harvest mitochondrial proteins.

### Measurement of mitochondrial DNA copy number, membrane potential, intracellular reactive oxygen species (ROS) and immunofluorescence

Mitochondrial DNA (mtDNA) copy number, membrane potential, ROS and immunofluorescence were analyzed and conducted as described 18,21.

### Animal experiments

Animal experiments were conducted as described 22. In brief, 8-week-old healthy male C57BL/6 mice were randomized into 6 groups with free access to water and food. Three of the 6 mice groups were fed with standard chow diet (CD, Purina 5001 from LabDiet with 14% kcal from fat) for 8 (CD-8), 12 (CD-12) or 16 (CD-16) weeks till the endpoint of experiment. The other 3 groups were fed with high-fat diet (HFD, from Research Diets D12492 with 60% kcal from fat, typically lard) for 8 (HFD-8), 12 (HFD-12) or 16 (HFD-16) weeks 22. Body weights (BW) and fasting biochemical parameters [glucose (GLU), total cholesterol (TCHOL) and TG] were continuously analyzed with the Fujifilm (Kanagawa, Japan) DRI-CHEM4000i. Serum TCHOL (Wako, Osaka, Japan), TGs (Stanbio Laboratory, San Antonin, TX), and GPT/GOT were determined using the Alanine Aminotransferase Activity (ALT/GPT)/Aspartate Aminotransferase Activity (AST/GOT) Assay Kit (MyBioSource, San Diego CA). Histological analysis and intra-peritoneal glucose tolerance test (ipGTT) were conducted as described 22, 23. Mice were sacrificed and liver were collected at each experimental endpoint. Target proteins in liver homogenate were probed by Western blotting. Animal protocols were reviewed and approved by the Institutional Animal Care and Use Committee, National Yang Ming Chiao Tung University (1041255).

### Statistical analysis

Each experiment was carried out at least three times. Results were presented as mean ± SEM and significant differences between groups were determined by two-tailed unpaired Student t-tests or two-way ANOVA with post hoc Tukey's test. Statistical significance was defined as *P* < 0.05 for all tests.

## Results

### Sequential alterations of energy metabolism and mitostasis in lipid-laden BNL cells

**Figure 1:**
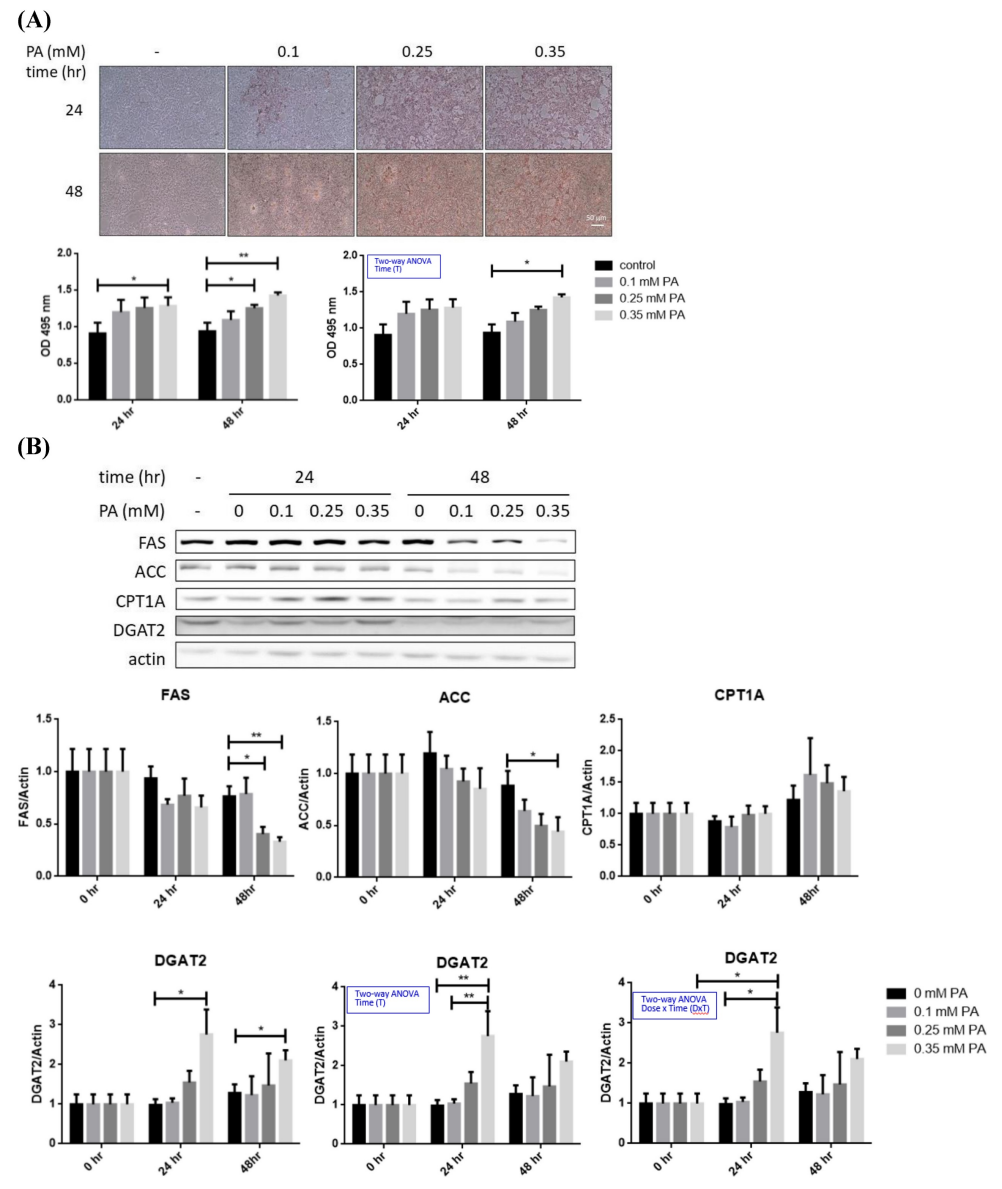

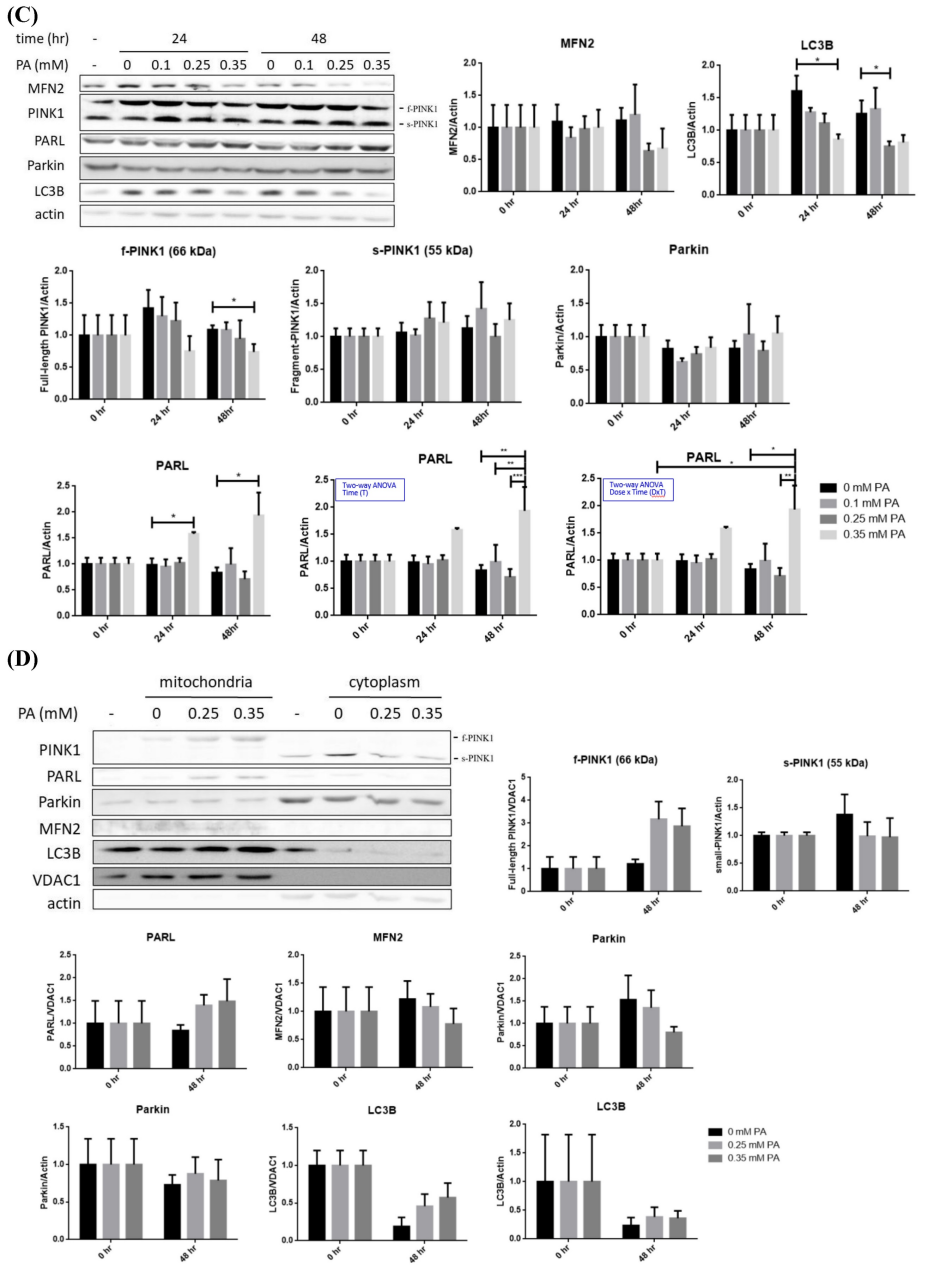

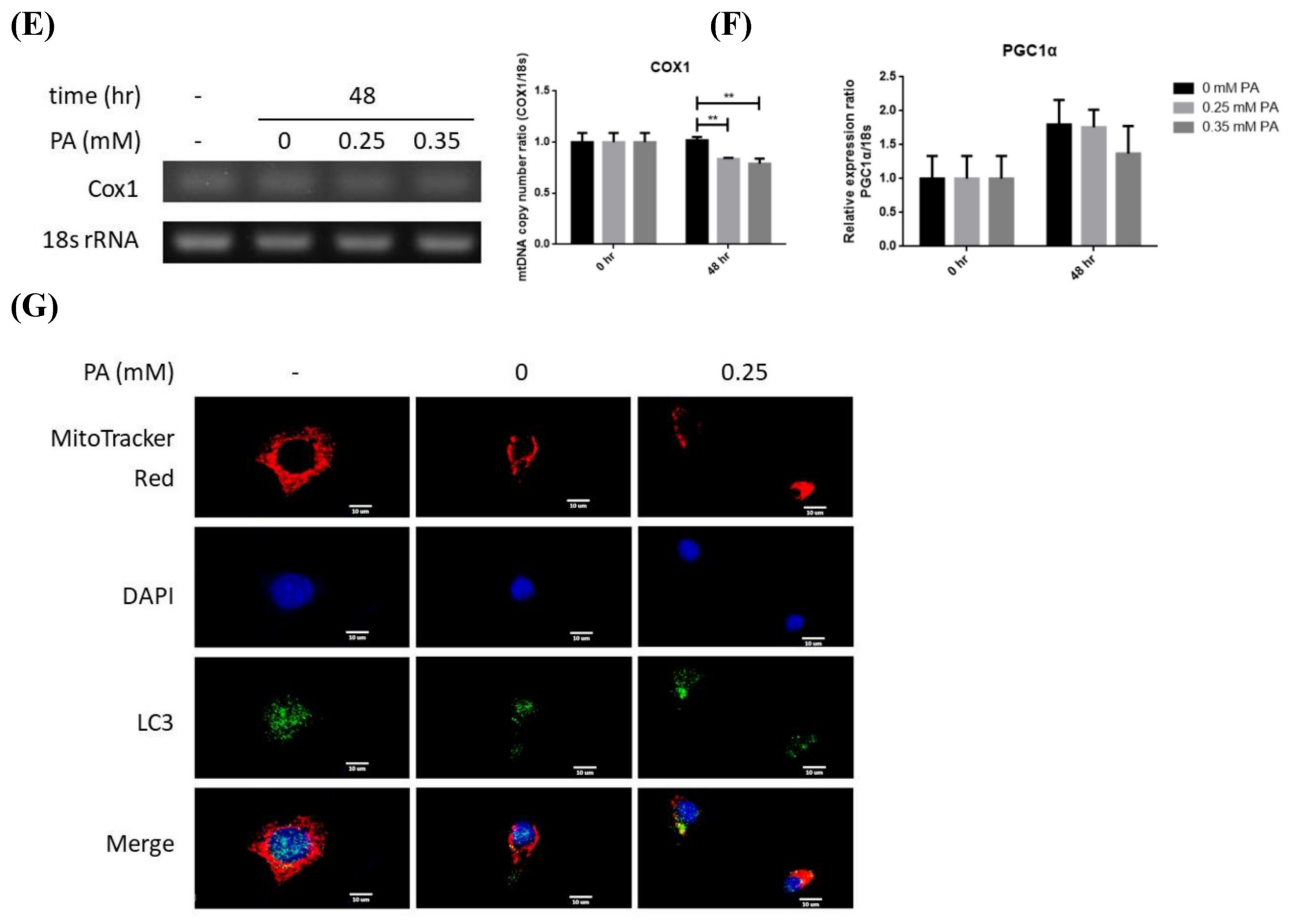
** Profiles of lipid metabolic and mitophagy-controlling machinery in lipid-laden BNL hepatocytes.** (A) Cells were treated with differential doses of palmitic acid (PA) as time indicated and subjected to ORO staining (n=5). (B) Levels of fatty acid synthase (FAS), acetyl coenzyme A carboxylase (ACC), diacylglyceride acyltransferase 2 (DGAT2) and carnitine palmitoyl transferase 1 A (CPT1A) were analyzed by Western blot (n=4). Expression (C) and distribution (D) of MFN2, PINK1, PARL and LC3B were analyzed by Western blot. (E) Cytochrome c oxidase I (COX1) levels were examined by PCR and normalized to 18s rRNA (n=3). (F) Peroxisome proliferator-activated receptor gamma coactivator 1-alpha (PGC1α) mRNA was analyzed by quantitative real-time PCR (n=4). Data were presented as the mean ± SEM, and statistically analyzed by two-tailed unpaired Student t-test or two-way ANOVA and post hoc compared by Tukey's test as indicated. ^*^P< 0.05 and ^**^ P< 0.01 vs control. (G) Confocal microscopy analysis showing colocalization of mitochondria and LC3B. All images were obtained with Lecia DM6000B standard upright fluorescence microscope (100X), scale bar=10 μm.

Imbalance of lipid metabolism is the major factor leading to hepatic steatosis 24. In this context, profile of critical lipid-metabolic enzymes was analyzed in BNL hepatocytes after lipid oversupply (**Figure 1A**). TG-synthesizing enzyme DGAT2 was significantly increased approximately 2 folds after 24 hr of PA exposure (**Figure 1B**), while FAS and ACC were significantly decreased (~50%) at 48 hr in a dose dependent manner. Alterations of CPT1A showed no significance. Therefore, PA oversupply rapidly promotes long-chain FA synthesis, followed by inhibiting de novo lipogenesis without affecting lipid oxidation. The net effect of FA oversupply is significantly higher hepatic adiposity via boosting TG synthesizing efficiency and the intracellular lipid storage (LDs; **Figure 1A**).

PARL was significantly increased about 1.5 folds while LC3B was significantly decreased at 24 hr, preceding the significant reduce of mitophagy inducer f-PINK1 at 48 hr (**Figure 1C**). The timeline of PARL elevation paralleled with that of f-PINK1 decrease. No significant differences concerning s-PINK1 and Parkin were observed. Cytosolic and mitrochondrial distribution of f-PINK and s-PINK1 was subsequenetly examined. Intriguingly, mitochondrial-docked f-PINK1 was increased under PA treatment while cytoplasmic s-PINK1 was decreased (**Figure 1D**). Mitochondrial surface-docked MFN2, Parkin and LC3B remained unchanged. The results imply that f-PINK1 and LC3B are translocated from cytoplasm to mitochondrial surface to elicit mitophagy in response to FA oversupply.

Enhanced mitophagy activity may lead to reduced mitochondrial mass, which is evidenced by the significantly lower copy number of COX1 at 48 hr (**Figure 1E**). Whereas, critical mitochondrial biogenesis-driving transcription factor PGC1α 25 was not prominently altered (**Figure 1F**). Therefore, lipid supply reduces mitochondrial mass without affecting mitochondrial biogenesis, suggesting mitophagy is the underlying factor leading to the decreased mitochondrial mass.

Nucleus-localized LC3B translocates to cytoplasm for autophagosome assembly after being activated by autophagy-inducing signals 26. Thus, LC3B localization was investigated. Reduced nuclear LC3B signal and cytoplamic co-localization of LC3B with mitochondria were identified at 48 hr (**Figure 1G**). The findings further support that lower mitochondrial mass is resulted from enhanced mitophagy rather than mitochondrial biogenesis.

Taking together, the sequential events of BNL cells under FA oversupply include the initial transiently up-regulated long-chain FA synthesis and increased lipid storage, followed by suppressed glucose metabolic efficacy and decresaed mitochondrial mass resulted from enhanced mitophagy. Whereas, lipid β-oxidation and mitochondrial biogenesis are not prominently affected.

### Alterations of energy metabolism and mitostasis in lipid-laden HepG2 cells

Cancer cells are metabolically re-programmed to preferentially produce energy via glycolysis rather than OXPHO 27. We, therefore, probed the metabolic alterations of HepG2 cells in response to FA oversupply.

**Figure 2:**
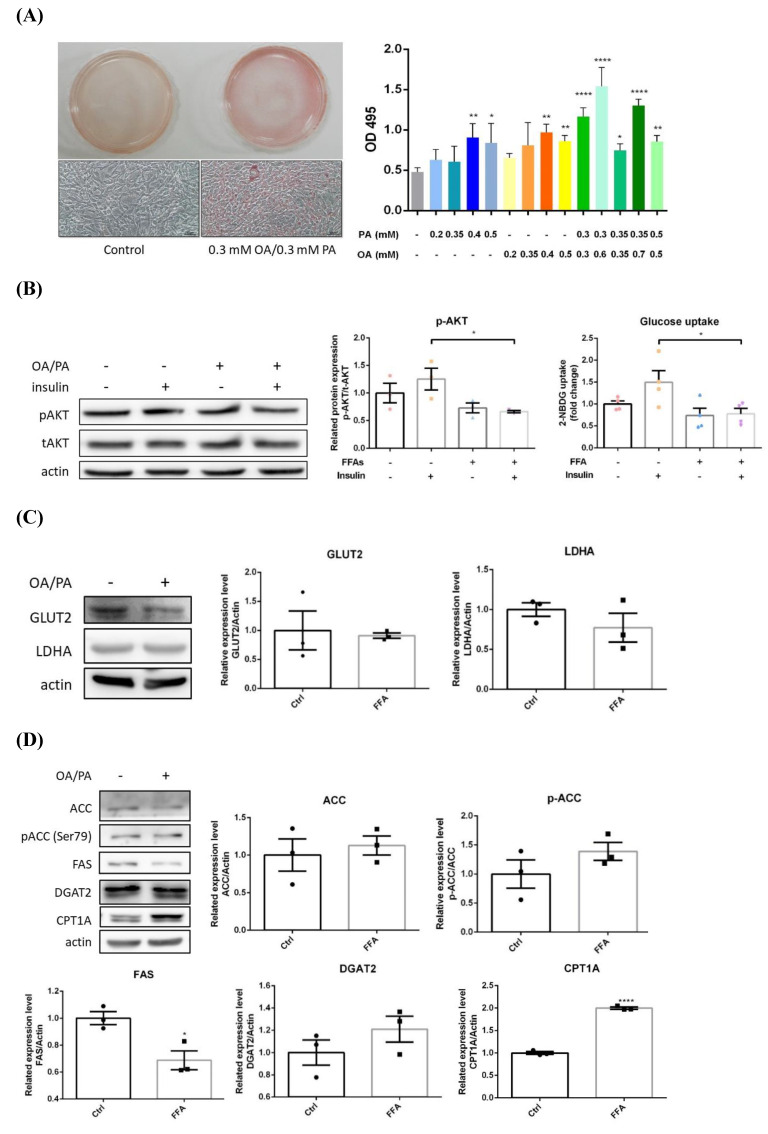

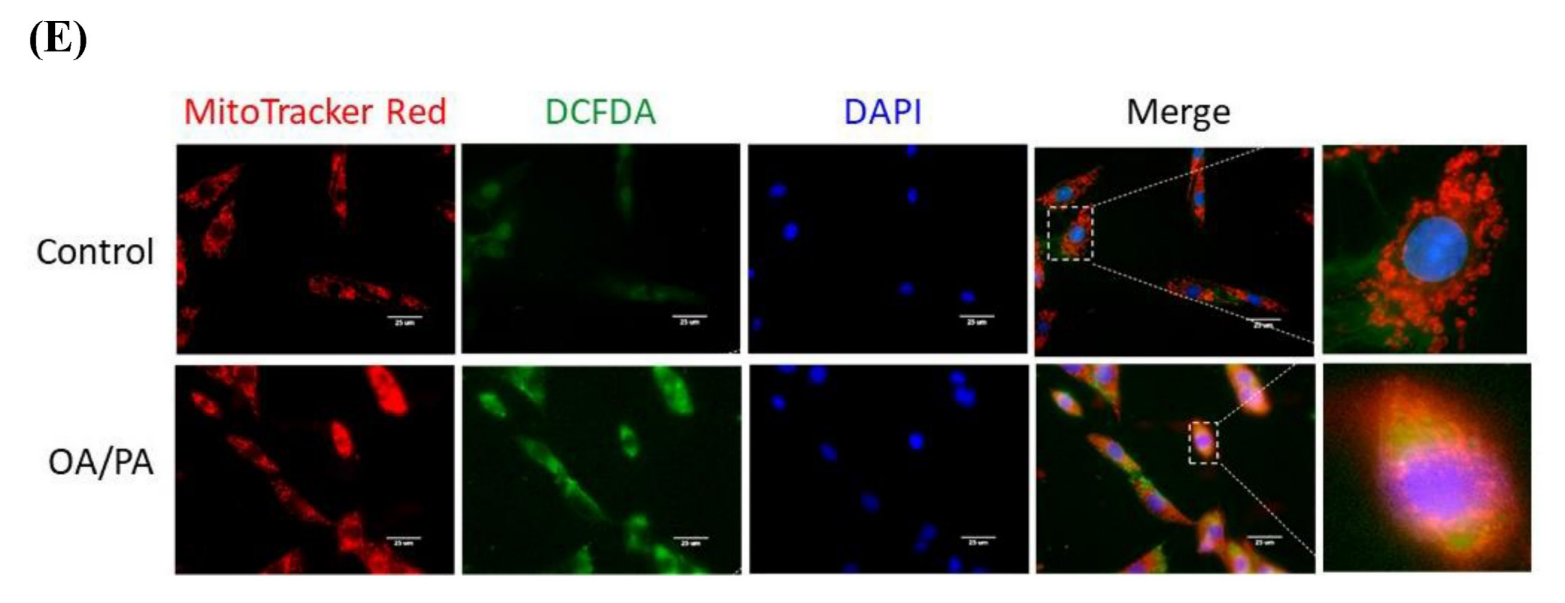
** Alterations of energy metabolism in lipid-laden HepG2 cells.** (A) Cells were treated with differential doses of OA and/or PA for 24 hr and subjected to ORO staining (n=3). Magnification: 200х. Scale bar=25 μm. (B) AKT/p-AKT and glucose uptake were respectively analyzed by Western blot and fluorescent glucose analog 2-NBDG (n=3). (C) GLUT2/ LDHA and (D) critical enzymes for lipid synthesis and β-oxidation were analyzed by Western blot (n=3). Data were presented as the mean ± SEM and statistically analyzed by two-tailed unpaired Student t-test. ^*^ P< 0.05, ^**^ P< 0.01 & ^****^ P< 0.01 vs control. (E) Intracellular reactive oxygen species (ROS) were analyzed by fluorescent H_2_DCF-DA and visualized by confocal microscopy. Magnification: 630х. Scale bar=25 μm.

After 24 hr of OA/PA co-treatment, significantly higher intracellular lipid contents were observed (**Figure 2A**). Insulin-induced pAkt and glucose uptake were significantly reduced about 40% and 30% respectively (**Figure 2B**), indicating insulin efficacy and glucose uptake were rapidly impaired. Glucose transporter 2 (GLUT2) and lactate dehydrogenase A (LDHA) were slightly decreased, however, with no statistical significance **(Figure 2C)**. Pro-lipogenic pACC/ACC were slightly increased while FAS was significantly inhibited about 30%, and DGAT2 was not substantially affected (**Figure 2D**). In contrast to BNL cells, CPT1A was significantly elevated about 2 folds. Hence, glucose-derived *de novo* lipogenesis is inhibited, whereas, lipid β-oxidation is promoted without affecting TG synthesis. Massive energy needs of cancer cells for supporting rapid proliferation is very likely the reason underlying the above findings. The transformation of filamentous mitochondrial network into fragmented puncta and significantly increased intracellular ROS strongly suggest mitochondrial dysfunction (**Figure 2E**). Notably, the induced lipid β-oxidation and morphological alterations are distinctive features from BNL cells. Accordingly, HepG2 cells exhibit lower insulin and glucose metabolic efficacy, as well as flattening and elongation of cell morphological changes under ambient FA supply.

**Figure 3:**
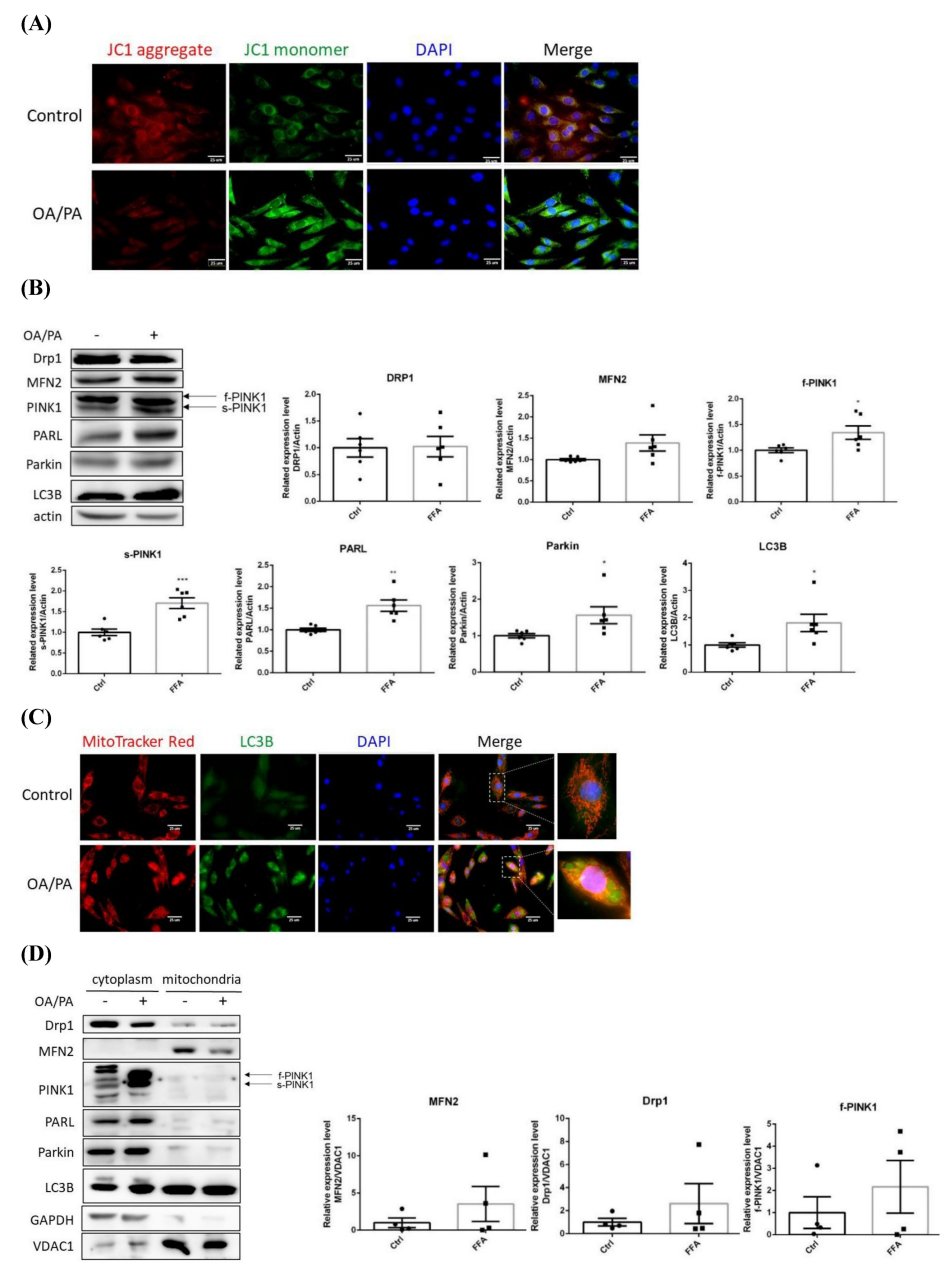

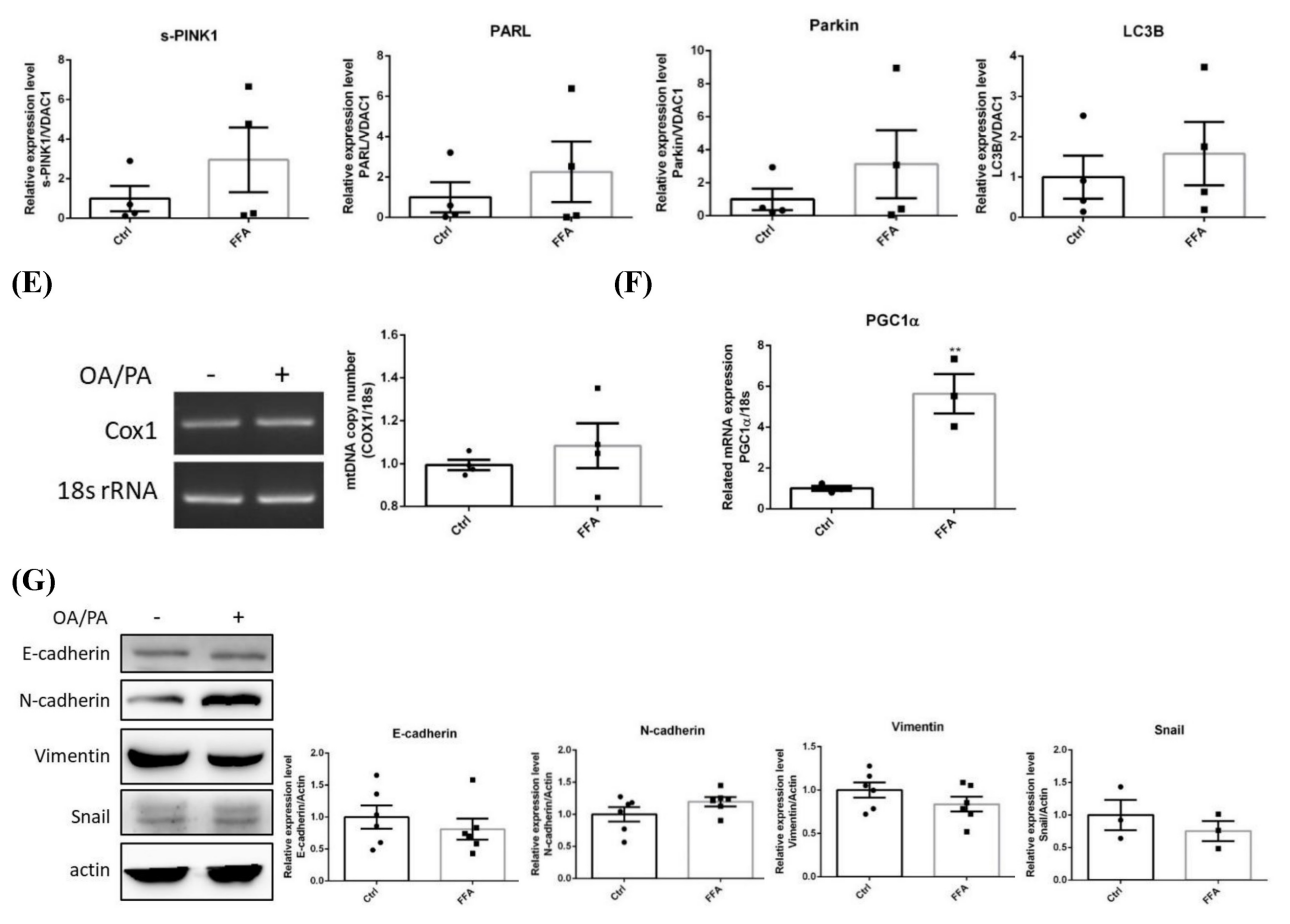
** Alterations of mitochondrial membrane potential, mitophagy-controlling machinery and epithelial-mesenchymal transition markers of lipid-laden HepG2 hepatocytes.** Confocal microscopic images showing (A) mitochondrial membrane potential analyzed by fluorescent JC-1 dye and (C) distribution of mitochondria and LC3B. Image magnification: 630х. Scale bar=25 μm. Expression (B) (n=6) and distribution (D) (n=4) of critical proteins controlling mitochondrial quality analyzed by Western blot. (E) Mitochondrial DNA cytochrome c oxidase I (COX1) copy number analyzed by PCR normalized to 18s rRNA (n=3). (F) PGC1α mRNA analyzed by quantitative real-time PCR (n=4). (G) Levels of E-cadherin, N-cadherin, vimentin and Snail analyzed by Western blot analysis. Data were presented as the mean ± SEM, and statistically analyzed by two-tailed unpaired Student t-test. ^*^ P< 0.05, ^**^ P< 0.01, and ^***^ P< 0.001 vs control.

The disturbed mitochondrial membrane potential demonstrated that mitochondrial function was greatly affected (**Figure 3A**), in addition, the original spindle cells were flattened and elongated to become more fibroblast-like shape. Drp1 and MFN2 remained consistent while PINK1 (both f-PINK1 and s-PINK1), PARL, Parkin and LC3B were all significantly elevated (**Figure 3B**). Cytoplasmic LC3B was significantly enhanced and co-localized with mitochondria puncta (**Figure 3C**). Mitochondria-docked f-PINK1, s-PINK1, Parkin, PARL and LC3B tended to be increased, however, without significant difference (**Figure 3D**). Intriguingly, OA/PA induced a prominent shift of multiple cytoplasmic PINK1 expression profile to the predominant expression of f-PINK1 and s-PINK1. While COX1 remained consistent (**Figure 3E**), PGC1α was signficantly increased about 5 folds (**Figure 3F**). Therefore, mitophagy machinery is recruited on mitochondrial surface to elicit mitophagy for eradicating dysfunctional mitochondria. Nevertheless, compensatory mitochondrial biogenesis is boosted to maintain mitochondrial mass for efficiently generating ATP to support cell proliferation.

NFALD is one of the risk factors of HCC incidence. Putative induction of epithelial-mesenchymal transition (EMT) by lipids was next analyzed as the above imaging data showed that FA triggers spindle to fibroblastic morphological changes. Interestingly, E-cadherin was reduced about 20% while N-cadherin was increased about 20% (**Figure 3G**). Thus, cell phenotypic transition from oval-spindle to elongated fibroblastic shape with higher plasticity is suggested to be elicited by the lipid-potentiated EMT.

### Sequential alterations of energy metabolism and mitostasis in high fat diet-induced NAFLD mice

**Figure 4:**
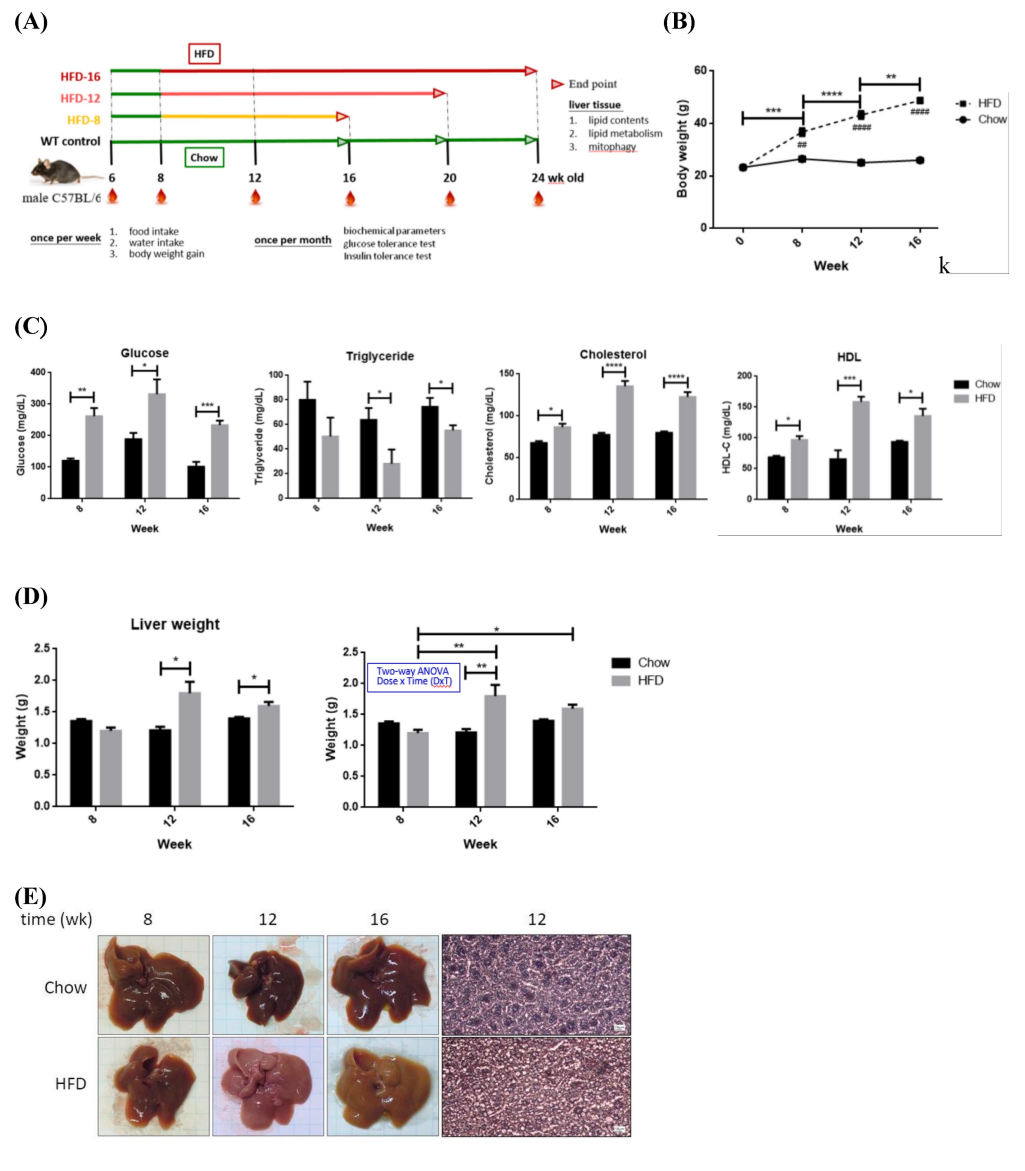

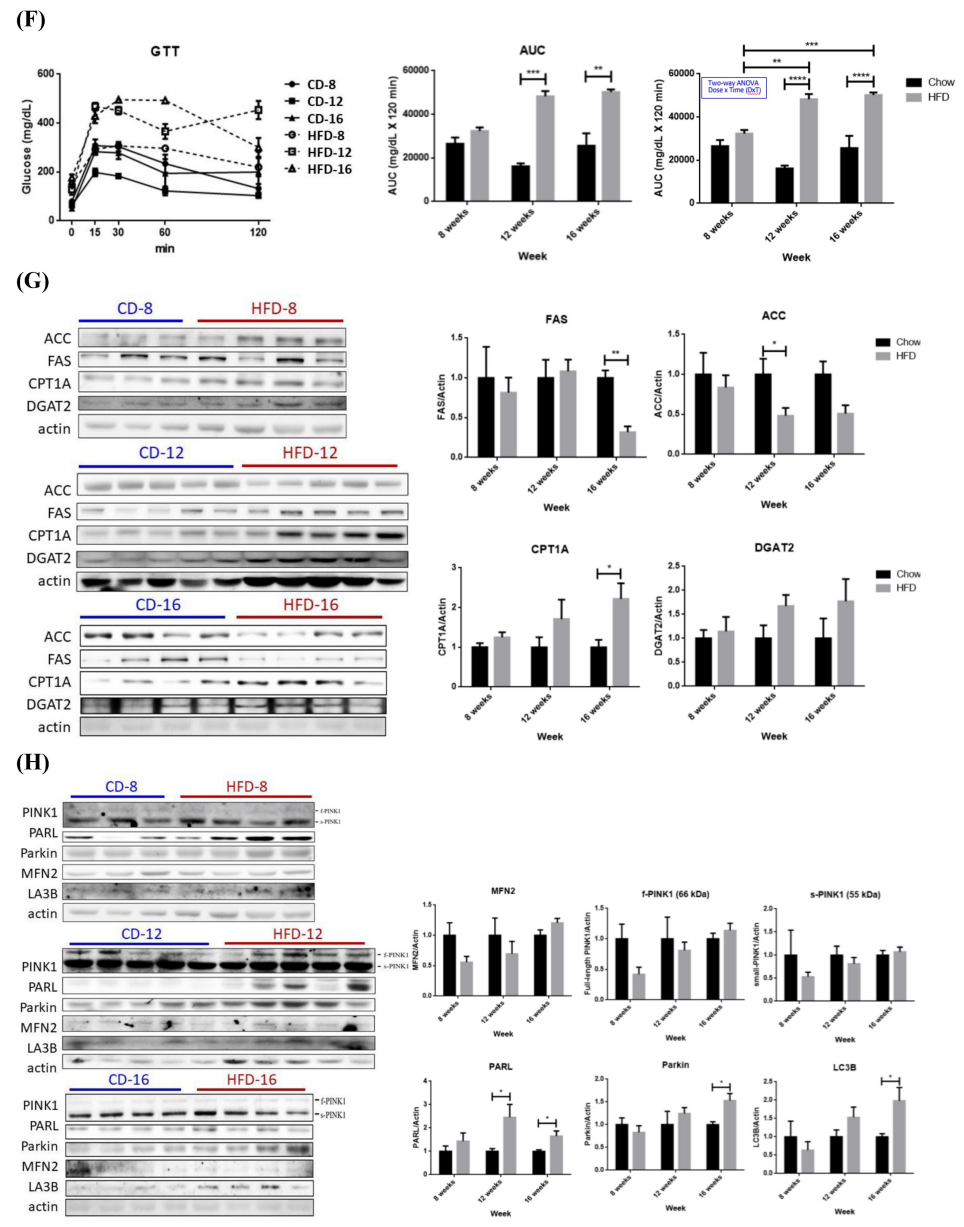
** Pathophysiological features and expression profile of critical enzymes for fatty acid synthesis and oxidation from Chow- and HFD- feeding mice.** (A) Schematic diagram showing the experimental protocol for mice fed with standard chow diet (CD) or high fat diet (HFD) for the indicated period. (B) Body weights and (C) serum glucose, triglyceride, cholesterol and HDL were measured at the indicated time. (D) Liver weights of all mice groups were recorded at the indicated time. (E) Hematoxylin and eosin histochemical staining showing significantly increased lipid contents in liver harvested from HFD-12 mice. (F) Intra-peritoneal GTT (ipGTT) was performed by injecting mice with glucose (2 g/kg). ^*^ P< 0.05, ^**^ P< 0.01, ^***^ P< 0.001 and ^****^ P< 0.0001 vs the corresponding control; ^##^ P< 0.01 and ^####^ P< 0.0001 vs the corresponding Chow. Hepatic expression profile of (G) FAS, ACC, DGAT2 and CPT1A and (H) MFN2, PINK1, PARL, Parkin and LC3B were temporally analyzed by Western blot. Data were presented as the mean ± SEM (n=3~5), and statistically analyzed by two-tailed unpaired Student t-test or two-way ANOVA and post hoc compared by Tukey's test as indicated. ^*^ P< 0.05 and ^**^ P< 0.01 vs Chow.

NAFLD animal models were established for characterizing the timeline of FA oversupply-induced metabolic events. Eight-week old mice were fed with either chow (WT control) or high-fat diet (HFD) for differential periods **(Figure 4A)**. BW, GLU, TCHOL and HDL of HFD mice were significantly elevated **(Figure 4, B~C)**. HFD-12 and HFD-16 mice had significantly higher liver mass **(Figure 4D)** due to elevated adiposity **(Figure 4E)**. For ipGTT **(Figure 4F)**, blood glucose of CD mice peaked around 200~300 mg/dL after 15~30 min of glucose infusion then returned to normal within 120 min. Glucose of HFD-12 and HFD-16 mice reached climax at ~450 mg/dL during 15~30 min and remained high thereafter till 120 min. Therefore, HFD-12 and HFD-16 mice showed impaired glucose tolerance by having significantly higher ipGTT AUC (*P*< 0.005 for HFD-12 and *P*< 0.01 for HFD-16). Notably, the glucose tolerance was exacerbated along with the duration of HFD consumption. In brief, HFD-8 mice exhibited normal physiological and biochemical parameters except for the significantly increased BW. On the contrary, HFD-12 mice manifested glucose intolerance, metabolic imbalance and the characteristic NAFLD hepatic lipid overload.

Hepatic expression profile of genes involved in lipid metabolism in HFD-8 mice were not significantly altered (**Figure 4G**). Significantly reduced ACC was first observed in HFD-12 mice, followed by lowered FAS and significantly elevated CPT1A in HFD-16 mice. Therefore, glucose metabolic efficiency is initially inhibited while lipid β-oxidation pathway is promoted by ambient lipid supply.

No significant alterations of mitophagy machinery were identified in HFD-8 mice **(Figure 4H)**. PARL was significantly increased in HFD-12 mice, prior to alteration of other mitophagy-associated proteins. MFN2, f-PINK1 and s-PINK1 remained consistent while PARL, Parkin and LC3B were significantly increased in HFD-16 mice. Therefore, significantly increased PARL and hepatic adiposity are first identified in HFD-12 mice, which are further boosted in HFD-16 mice.

In summary, mice consuming 12-wk HFD manifest glucose intolerance, metabolic imbalance and NAFLD symptom of increased hepatic adiposity. Ambient lipid supply tilts metabolic homeostasis to using lipids as the major energy source, which results in elevated lipid β-oxidation and suppressed glucose metabolic efficacy. Hepatic lipid overload elicits metabolic re-programming and thus triggers mitophagy activity.

## Discussion

### Temporal *in vivo* alterations of physiological parameters and mitostasis induced by high-fat diet

Physiologically, we characterized that 12-wk of HFD consumption is a critical time point of NAFLD development demonstrated by the increased liver mass, hepatic adiposity and impaired glucose tolerance **(Figure 4)**. Influx of circulating TGs to liver is suggested as the underlying reason for lower blood TGs in HFD-12 mice 11. Significant changes of hepatic ACC and PARL expression in HFD-12 mice are the earliest identified molecular events in response to lipid oversupply **(Figure 4)**, with *de novo* lipogenesis and glucose metabolic efficacy affected prior to mitostatic changes. Significantly reduced FAS and increased CPT1A indicate that HFD-16 mice preferentially metabolize FA. In brief, deterred glucose metabolic efficacy and enhanced PARL are the first alterations 18 which then mediate mitophagy by provoking mitochondrial quality-control machinery.

### Temporal *in vitro* alterations of energy metabolism and mitostasis in lipid-laden hepatocytes

#### Non-cancerous BNL cells

Long-chain FA synthesis alteration is the first identified metabolic change of BNL cells in response to FA oversupply, followed by the reduced *de novo* lipogenesis and increased lipid storage **(Figure 1)**. Negative feedback by the significantly elevated lipids storage after 24 hr of FA exposure is very likely to down-regulate the significantly increased DGAT2. A dose-dependent down-regulation of *de novo* lipogenic FAS and ACC is identified, leading to inefficient glucose metabolic efficacy and predisposing the cells to glucose intolerance.

In support of the *in vivo* findings and our previous study 18, PARL is the first up-regulated gene under lipid oversupply **(Figure 1)**. FA supplement induces a dose-dependent change of mitochondria-docked f-PINK1 and cytoplasmic s-PINK1, besides, the co-localization of LC3B and mitochondria suggest that mitophagy is triggered. Unaltered PGC1α suggests the reduced mitochondrial mass is caused by enhanced mitophagy, rather than decreased mitochondrial biogenesis. Therefore, sequential events of BNL cells in response to FA oversupply include the initial elevated long-chain FA synthesis and lipid storage, followed by suppressed glucose metabolic efficiency and mitophagy-mediated decreased mitochondrial mass.

#### HepG2 hepatic cancer cells

In HepG2 cells, FA oversupply rapidly disturbed insulin efficacy and glucose uptake **(Figure 2)**. Glucose metabolic efficacy and *de novo* lipogenesis are inhibited as the scenario in BNL cells, nevertheless, lipid β-oxidation is dramatically elevated 27. The increased ROS and transformation of filamentous mitochondrial network into fragmented puncta indicate mitochondrial function is greatly impaired. These dysfunctional mitochondria are then targeted to be eliminated by mitophagy. A compensatory boosted mitochondrial biogenesis, reflected by the increased PGC-1α, is identified for maintaining mitochondria mass and function.

### Distinctive features of cancerous and non-cancerous hepatocytes in environment with lipid oversupply

**Figure 5:**
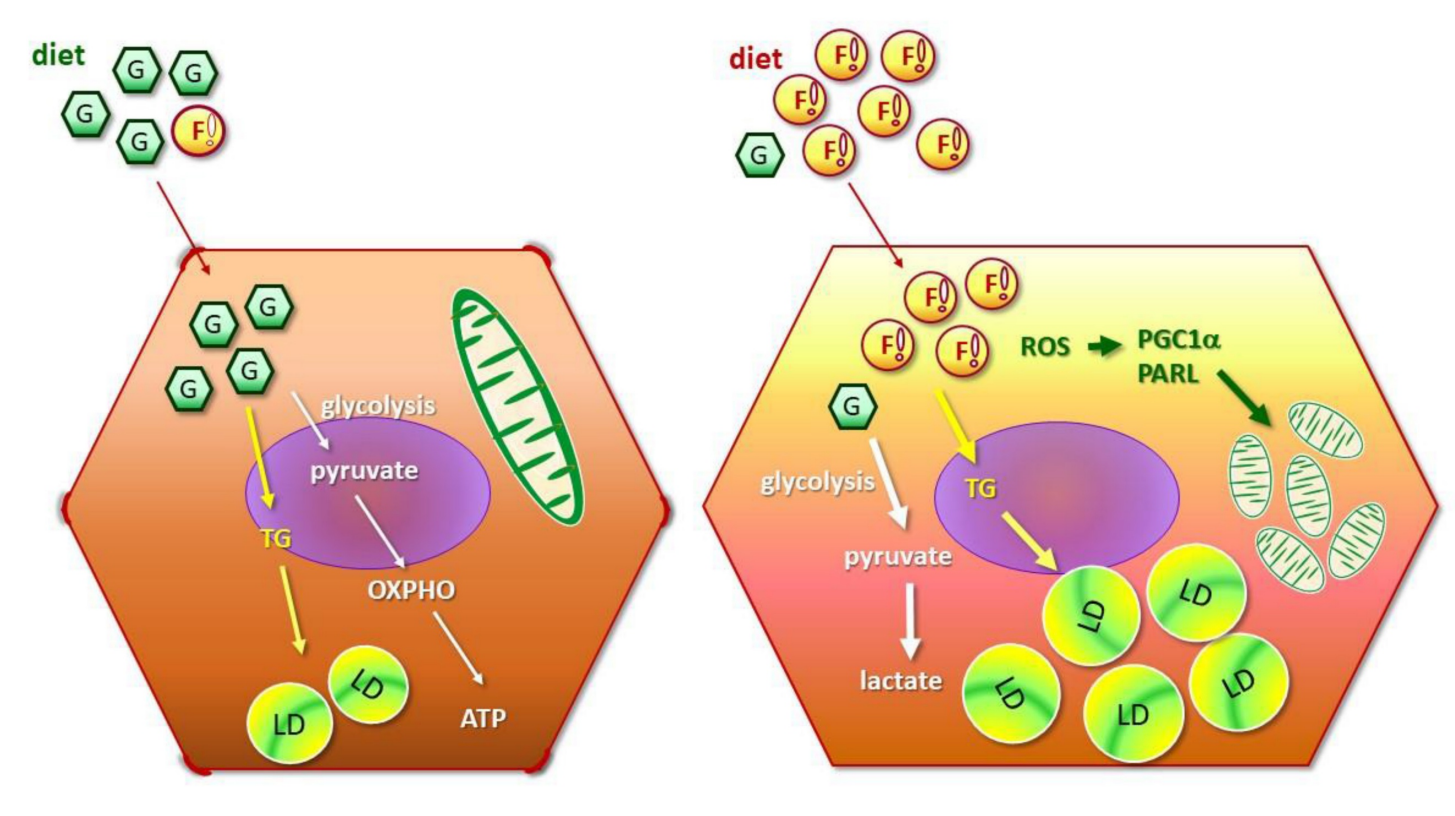
** Metabolic and mitostasis alterations of hepatic cancer cells in response to fatty acid oversupply.** FA triggers mitophagy elicitation, morphological changes from oval-spindle cell into elongated fibroblast-like phenotype and higher N-cadherin expression, which potentiates EMT and confers the cells with higher plasticity and metastatic capability. Mitochondrial stress, accumulated ROS and lipid peroxidation further boost cell proliferation and invasiveness, and thus aggravate malignant traits.

Suppressing glucose metabolic efficacy and *de novo* lipogenesis is the common effect of FA oversupply on BNL and HepG2 cells. Nevertheless, distinctive features of HepG2 cells in response to FA oversupply are characterized (depicted in **Figure 5**). First of all, lipid β-oxidation is boosted 28. Secondly, mitochondrial biogenesis is prominently enhanced as a compensatory feedback response, aiming at maintaining mitochondrial mass to generate sufficient energy for supporting rapid cellular growth. Thirdly, EMT potentiation and cell morphological changes are identified. In addition, insulin signaling and glucose uptake are rapidly deterred. PGC1 family proteins are powerful energy sensors that regulate mitochondrial biogenesis and energy metabolism 29. Distinct PGC1α profiles manifested by BNL and HepG2 cells under FA supply indicate that flexibility of mitochondrial adaptation is dynamically adjusted by metabolic status and corresponding energy demands.

Metabolic reprogramming is the well-characterized feature in malignant cells, designated as “Warburg effect” 30,31. Suppressed glucose uptake enables the cells adapt to nutrient-deprived and hypoxic conditions that the rapid growing cells are frequently encountered. Enhanced lipid β-oxidation generates ROS, leading to oxidative damage, genomic instability, and activation of pro-survival signals. The metabolic shift eventually results in inflammation and tumor progression 32. Collectively, these changes create a pro-tumorigenic metabolic environment characterized by dysregulated survival, proliferation, malignant transformation, eventually forming a vicious cycle which facilitates tumor progression and therapeutic drug resistance 33-35.

The present study not only echoes the above reports but also provides *in vitro* and *in vivo* evidence demonstrating the timeline concerning FA supply-facilitated metabolic imbalance and mitochondrial dynamics. Notably, FA also triggers flattening of oval-spindle cells into elongated fibroblast-like phenotype with higher N-cadherin, conferring the cells with higher plasticity and metastatic capability. Mitochondrial stress, accumulated ROS and lipid peroxidation further boost cell proliferation and invasiveness, and thus aggravate malignant traits 11. Accordingly, NAFLD progression is accelerated by the potentiated cellular EMT and higher flexibility which result in the predisposition of HCC development and onset.

### Implication of metabolic changes, ROS production, and mitostasis in physiological and pathological outcomes of hepatic diseases

Metabolic reprogramming also affects pathological processes and facilitates the transition of benign NAFLD into irreversible hepatic diseases via regulating mitostasis 36,37. The increased lipid β-oxidation and derived ROS under lipid oversupply lead to mitochondrial fission and dysregulated biogenesis which exacerbate hepatic steatosis 38,39. As fibrosis develops, mitophagy dysfunction and ROS-induced DNA damage further excerbate hepatic injury and fibrosis 40, 41. The impaired mitostasis supports oncogenic metabolism, which promotes mitochondrial fragmentation, genomic instability, and tumorigenesis 39,42,43. Furthermore, chemotherapy-induced mitophagy helps HCC cells remove damaged mitochondria, thereby increasing resistance to anti-cancer drug-induced apoptosis 44-46. These studies underscore the importance of mitochondrial dynamics and dysfunction in liver disease progression. Accordingly, targeting mitochondrial dynamics offers a promising therapeutic approach to mitigate NAFLD progression to HCC.

### Role of mitophagic biomarkers PINK1/LC3B in mitostasis and cell damage

Mitochondrial membrane potential (mMP) is essential for ATP synthesis via OXPHO in electron transport chain (ETC), generating ROS as by-products. In general, a high mMP reflects healthy mitochondria while loss of mMP (depolarization) impairs ETC, increased ROS and impaired ATP production 47. Therefore, persistent depolarization and ROS generation are hallmarks of mitochondrial dysfunction.

Mitophagy is elicited for removing damaged mitochondria via triggering the mitochondrial quality control keeper PINK1 when the cells encounter mitochondrial depolarization and elevated ROS 18,47,48. After being activated, the outer mitochondrial membrane (OMM)-accumulated PINK1 recruits Parkin to elicit the mitophagy cascade of OMM protein ubiquitination, LC3B-mediated autophagosome formation and mitophagic engulfment 49. In brief, mitochondrial ROS production and membrane depolarization are tightly correlated with mitochondrial dysfunction and mitophagy 47,48. Cells may proceed to apoptosis or necrosis if mitophagy fails to mitigate cellular damage due to excess ROS or irreversible depolarization 50. Thus, the balance of these above signals is crucial for maintaining mitostasis and cellular survival 49. In this context, thorough understanding the role of dysregulated mitochondrial dynamics in hepatocyte transformation as well as deciphering the underlying molecular mechanisms is critical for identifying putative targets and paving the way to develop novel therapeutic strategies.

### Physiological relevance of the experimental lipid conditions

#### *In vitro* OA/PA treatment

PA and OA concentrations in metabolic research are mostly 0.2~0.5 mM and 0.5~1.0 mM respectively 51,52, while physiological and pathophysiological plasma FA levels in humans vary depending on individual metabolic state. In general, the fasting plasma FA reference range 0.3-0.6 mM would rise to 0.8-1.0 mM or higher in obese or insulin-resistant individuals 53-55. PA comprises about 20-30% (~0.06-0.2 mM) of the total plasma FA and is often metabolized to produce toxic lipids which result in lipotoxicity. On the other hand, OA comprises approximately 40-50% (~0.12-0.5 mM) of the total plasma FA and is generally protective to mitigate palmitate toxicity. The OA/PA treatment conditions in the study were established for the cells to reach maximal intracellular lipid deposits and cell viability after a series of dose response and time course pre-tests were conducted.

#### *In vivo* high fat diet conditions

HFD-induced obesity is the most commonly used strategy in NAFLD study. Fat composition in the standard CD and HFD are generally 10% and 60%, respectively. CD formula is much closer to the recommended human health diet and more nutritionally balanced than HFD. Although HFD-fed mice manifest similar metabolic disease progression to human, differences in diet composition, genetic homogeneity, and the absence of co-morbidities limit the translational relevance. In particular, a big gap exists between the well-controlled experimental conditions in laboratory and the real complicated socio-environmental and lifestyle pathogenic factors linking to human NAFLD onset and progression 56,57.

Despite of these limitations, findings from HFD-fed mice model provide valuable information and clues concerning potential therapeutic targets and biomarkers for NAFLD. As impaired mitophagy and mitochondrial dysfunction are implicated in NAFLD, targeting PINK1/Parkin pathway to restore mitochondrial quality 58, the disrupted AMPK/PPARα signaling-resulted enhanced β-oxidation 59, dysregulated ER stress 58, PI3K/Akt signaling cascade and inflammatory responses 59 are reported as potential therapeutic strategies. Nevertheless, the translation value of these findings into human clinical studies still requires meticulous validation.

### Significance and necessity of delineating sequential effects of FA oversupply on hepatocytes during NAFLD pathogenesis and disease progression

Impaired mitophagy is believed to take part in the pathogenesis of various diseases 60. Although declined mitochondrial function in NAFLD is generally recognized as a facilitative factor to NASH and HCC development, the heterogeneous alterations of mitostasis in NAFLD progression add substantial difficulties in justifying appropriate intervention timing and measures 11. In addition, EMT and energy metabolism are mutually and dynamically regulated during NAFLD progression 36. EMT-modulated metabolic adaptations grant cancer cells the energy for sustaining rapid growth 37. On the other hand, as shown in Figure 2 and Figure 3, metabolic alterations contribute to EMT which endows the cells with higher plasticity and metastatic properties. Thus, cancer metastasis can be potentially repressed via manipulating metabolic pathways to suppress EMT; vice versa, targeting EMT in metabolically-impaired cells attenuates tumor metastasis. As NAFLD manifestations represent net effects from the interaction among metabolic re-programming, mitostasis and EMT, better understanding the sequential events of hepatocytes in response to FA oversupply is the pre-requisite to develop effective therapeutic strategy 34,60.

NAFLD patients are generally encouraged to reduce body weight by dietary restrictions and increasing physical activity due to the lack of NAFLD- and NASH- specific treatment. Unfortunately, few patients can insistently stick to the beneficial healthy lifestyle. Thus, pharmacological interventions treating hyperlipidemia, insulin resistance and hyperglycemia are empirically prescribed in case of needs. Therefore, better understanding the molecular mechanisms underlying the accumulation of lipids, ROS, and liver fibrosis is essential to reduce the onset of hepatic steatosis.

Nevertheless, the timeline of hepatic adaptations under FA overload during NAFLD development and progression is not fully illustrated as the data from most of reports are cross-sectional studies. Therefore, it is challenging to elucidate the whole scenario of NAFLD disease progression. This study is designed to address temporal alterations and adaptations of hepatocytes under FA oversupply by *in vivo* and *in vitro* strategies. Our findings support Rector et al. 61 that liver cells preferentially use FA as energy resources under elevated FA influx. Intriguingly, Kwapisz et al. 62 revealed that 9-day prolonged exposure of FAs significantly activates inflammation and EMT in hepatocytes. In addition, EMT and hepatic fibrosis are significantly promoted in mice with 20-week HFD consumption. Taken their findings 61,62 and our data together, a clear picture of sequential cellular responses induced by FA oversupply are disclosed: an initial down-regulated long-chain FA synthesis and lipid storage, followed by suppressed glucose metabolic efficacy, increased lipid turnover which induces ROS and elevated mitophagy, as well as the EMT. Mitochondrial mass remains unchanged due to the compensatory boosted mitochondrial biogenesis. Notably, our data document the morphological and molecular changes concerning mitochondrial dynamics in terms of membrane potential, mitochondrial quality control machinery, mitophagy and mitochondrial mass **(Figure 3)**. In particular, the imaging data and reciprocal E- and N- cadherin expression reveal EMT changes. In brief, FA exposure results in metabolic deviations, dysregulated mitostasis, induced EMT and phenotypic changes which facilitate tumorigenesis and metastasis.

## Conclusion

As depicted in **Figure 5**, our data reveal that long-chain FA synthesis and lipid storage are first induced by FA oversupply, followed by suppressed glucose metabolic efficiency and decreased mitochondrial mass. Intriguingly, distinctive features of cancer cells in response to FA oversupply are characterized. In brief, insulin signaling and glucose uptake are rapidly deterred, lipid β-oxidation and mitochondrial biogenesis are up-regulated. The FA-induced mitophagy, cell morphological transition and higher N-cadherin expression potentiates EMT which confers the cells with higher morphological plasticity. Accordingly, NAFLD disease progression is accelerated to predispose the patients to HCC development. However, whether the identified timeline and hepatic physiological alterations induced by FA oversupply parallel with the clinical manifestations of human subjects is the limitation of our study and awaits further investigation. In particular, the disease progression of NAFLD varies among individuals. Nevertheless, this study provides evidence elucidating the sequential events caused by FA oversupply, moreover, delineates the facilitative role of lipid overload in shaping the environment for NAFLD hepatocytes to acquire malignant traits and evolve into irreversible consequences.

Given the rapidly increasing global prevalence of metabolic disorders and the heterogeneous NAFLD manifestations during disease progression, appropriate intervention timing and strategy are critical for ameliorating and/or reversing NAFLD progression since there is no return once the line between the benign NAFLD and irreversible NASH and HCC consequences is crossed. Accordingly, it is of great significance to better understand the sequential events which aid in identifying promising targets and developing tailor-made treatment for NAFLD patients. In summary, this study provides compelling evidence dissecting the evolution of FA oversupply-induced metabolism, mitophagy and EMT which, hopefully, is valuable reference in tackling this globally prevalent health threat.

## Abbreviations

ACC: acetyl coenzyme A carboxylase
ALT/GPT: alanine aminotransferase
AST/GOT: aspartate aminotransferase
AUC: area under curve
CD: chow diet
C/EBPα: CCAAT-enhancer-binding protein-alpha
COX1: cyclooxygenase-1
CPT1A: carnitine palmitoyl transferase 1A
DGAT2: diacylglyceride acyltransferase 2
EMT: epithelial-mesenchymal transition
FA: fatty acid
FABP4: fatty acid-binding protein 4
FAS: fatty acid synthase
FFAs: free fatty acids
f-PINK1: full-length PINK1
GLU: glucose
H2DCF-DA: 2',7'-dichlorodihydrofluorescein diacetate
HCC: hepatocellular carcinoma
HFD: high-fat diet
ipGTT: intra-peritoneal glucose tolerance test
LDs: lipid droplets
MFN: mitofusin
mtDNA: mitochondrial DNA
NAFLD: non-alcoholic fatty liver disease
NASH: nonalcoholic steatohepatitis
ORO: Oil-Red O
OXPHO: oxidative phosphorylation
PARL: presenilin-associated rhomboid-like protein
PINK1: PTEN-induced kinase 1
s-PINK1: small PINK1 fragment
PPARγ: peroxisome proliferator-activated receptor-gamma
ROS: reactive oxygen species
T2DM: type 2 diabetes mellitus
TGs: triglycerides
TCHOL: total cholesterol
VDAC: voltage-dependent anion-selective channel
